# Androgen Metabolism Gene Polymorphisms, Associations with Prostate Cancer Risk and Pathological Characteristics: A Comparative Analysis between South African and Senegalese Men

**DOI:** 10.1155/2012/798634

**Published:** 2012-10-02

**Authors:** Pedro Fernandez, Charnita M. Zeigler-Johnson, Elaine Spangler, André van der Merwe, Mohamed Jalloh, Serigne M. Gueye, Timothy R. Rebbeck

**Affiliations:** ^1^Department of Urology, Stellenbosch University, P.O. Box 19063, Cape Town 7505, South Africa; ^2^Department of Biostatistics and Epidemiology and Abramson Cancer Center, Perelman School of Medicine, University of Pennsylvania, Philadelphia, PA 19104, USA; ^3^Department of Urology, Tygerberg Hospital, Cape Town 7505, South Africa; ^4^University Cheikh Anta Diop and Hôpital Général de Grand Yoff, Dakar, Senegal

## Abstract

Prostate cancer is the most common cancer in men in developed countries and the leading cause of mortality in males in less developed countries. African ethnicity is one of the major risk factors for developing prostate cancer. Pathways involved in androgen metabolism have been implicated in the etiology of the disease. Analyses of clinical data and *CYP3A4*, *CYP3A5*, and *SRD5A2* genotypes were performed in South African White (120 cases; 134 controls), Mixed Ancestry (207 cases; 167 controls), and Black (25 cases; 20 controls) men, as well as in Senegalese men (86 cases; 300 controls). Senegalese men were diagnosed earlier with prostate cancer and had higher median PSA levels compared to South African men. Metastasis occurred more frequently in Senegalese men. Gene polymorphism frequencies differed significantly between South African and Senegalese men. The *CYP3A4* rs2740574 polymorphism was associated with prostate cancer risk and tumor aggressiveness in South African men, after correction for population stratification, and the *SRD5A2* rs523349 CG genotype was inversely associated with high-stage disease in Senegalese men. These data suggest that variants previously associated with prostate cancer in other populations may also affect prostate cancer risk in African men.

## 1. Introduction

Prostate cancer is the most common cancer in men from industrialized developed countries, and worldwide, the second most common of all malignancies in men [1–3]. The highest rates of prostate cancer are observed in African-American men in the United States of America (USA) and Caribbean men of African descent [1, 4], while the highest disease-associated mortality rates are observed in less developed countries that include regions of the Caribbean, Sub-Saharan Africa, and South America [3]. These data lend support to the suggestion that the African ethnicity is one of the major risk factors for prostate cancer [5, 6]. Although comprehensive cancer registries are limited in Africa, available data indicate that prostate cancer accounts for approximately 10.6% and 4.8% of all cancers in males in Sub-Saharan Africa and North Africa, respectively [7]. 

In Southern Africa (South Africa) and Western Africa (Nigeria and Cameroon), prostate cancer is the most commonly diagnosed cancer in males; however, the incidence of prostate cancer in Southern Africa is double that observed in Western Africa [8]. The reported age-standardized rate of histologically diagnosed prostate cancer in South Africa is 74.4 per 100000 males in the White population (European ancestry), 48 per 100000 males in the South African Mixed Ancestry population (a unique population group of Khoisan, European, African, and Asian ancestry that emerged in the 1700–1800 s; sometimes referred to South African Coloured) [9], and 17.2 per 100000 males in the Black population (African ancestry) [10]. However, the low reported incidence rate in South African Black men may be due to underreporting and underdiagnosis as a result of poor access to medical facilities [11]. Additionally, South African Black men present with a higher-grade and -stage disease, have higher serum prostate specific antigen (PSA) levels and receive potentially curative treatment less often than White or Mixed Ancestry men [12].

The clinical characteristics of prostate cancer among Senegalese men were found to be different from African-American and Caucasian-American men [13]. Additionally, Senegalese men were most often diagnosed later with prostate cancer-related symptoms and at a worse tumor stage [13]. Senegalese and Asian-Indian men were also shown to be more likely to present with advanced disease compared to African-American and Caucasian-American men [14]. A more recent investigation determined that prostate cancer had a prevalence of 3.8% in Senegalese men [15]. The same study also reported that prostatic intraepithelial neoplasia (PIN), which is considered to be a precancerous lesion [16], was detected in 29.1% of men in their study population. 

Because of the known hormone dependence of prostate cancer, genetic alterations in androgen metabolism pathways are likely to play a role in conferring genetic susceptibility to the disease. The androgen metabolism genes *CYP3A4* and *CYP3A5*, which encode proteins belonging to the cytochrome P450 (CYP) family of enzymes that are involved in the metabolism of xenobiotics, steroids, vitamins, and sex hormones, have been implicated in prostate cancer risk [17–21]. Other studies have demonstrated associations between polymorphisms in the steroid 5-alpha reductase gene (*SRD5A2*), which encodes an enzyme that converts testosterone to dihydrotestosterone (DHT), and the risk of developing prostate cancer or disease severity [22–26]. We previously described genetic associations between polymorphisms in androgen metabolism genes, and risk of developing prostate cancer in South African men [27] and reported differences in genotype and allele frequencies between Senegalese, African-American, Caucasian-American and Ghanaian men for *CYP3A4* and *SRD5A2* [28]. In the present study, we extended our previous investigations by including additional participants and compared clinical information and genotype data for polymorphisms in *CYP3A4*, *CYP3A5,* and *SRD5A2* between South African and Senegalese men. We describe differences in age at diagnosis, PSA levels, and metastasis in South African and Senegalese men. In addition, we show that genotype and allele frequencies in androgen metabolism genes differ between South African and Senegalese men and report on genetic associations with prostate cancer risk and disease aggressiveness.

## 2. Materials and Methods

### 2.1. South African Study Population

Categorization of all the study participants into different ethnic groups was based on self-identity. The study population comprising 120 White cases, 207 Mixed Ancestry cases, and 25 Black cases with histologically confirmed prostate cancer and no prior cancer at any other site were recruited from the Department of Urology, Tygerberg Hospital (Cape Town, South Africa). Clinical characteristics including PSA, Gleason grade, tumour node metastasis (TNM) stage, age at diagnosis, and other cancer diagnoses were obtained from medical records. All cases underwent radical prostatectomy, transurethral resection of the prostate, or prostatic biopsy and had histologically confirmed prostate cancer. Controls were selected among subjects admitted to Tygerberg Hospital for routine PSA examinations or benign prostatic hyperplasia (BPH) and comprised of 134 White men, 167 Mixed Ancestry men, and 20 Black men that were matched for ethnicity (self-reported) and were from the same geographical region. Inclusion criteria for controls were PSA levels of ≤4.0 ng/mL and a normal digital rectal examination (DRE). Four Mixed Ancestry men with a PSA ≥4.0 ng/mL and/or an abnormal DRE, but who were negative for prostate cancer upon histological examination, were included as controls. Individuals with a prior diagnosis of cancer at any other site were excluded from the study. 

### 2.2. Senegalese Study Population

The study population comprising 86 cases with histologically confirmed prostate cancer, advanced disease status with prostatic presentation at DRE or the existence of metastasis highly suggestive of prostate cancer, and with no prior cancer at any other site, were recruited from the Hôpital Général de Grand Yoff (Dakar, Senegal) and had undergone radical prostatectomy, transurethral resection of the prostate, or prostatic biopsy. A standardized questionnaire and review of medical records were used to obtain clinical information relevant to the study. Hospital and community-based controls were comprised of 300 men from the same geographical region. Inclusion criteria for Senegalese controls were PSA levels ≤4.0 ng/mL and normal DRE, or negative histology for prostate cancer. Individuals with a prior diagnosis of cancer at any other site were excluded from the study. 

All study subjects consented to participate in the study and allowed their biological samples to be genetically analyzed, according to the Declaration of Helsinki (1964). The study was approved by the Stellenbosch University Health Research Ethics Committee, and the Institutional Review Boards of the Hôpital Général de Grand Yoff and the University of Pennsylvania.

### 2.3. Genotyping Analysis

Blood and buccal swabs were collected from South African and Senegalese men, respectively. Genomic deoxyribonucleic acid (DNA) was extracted from all samples using the QIAamp DNA Blood kit (Qiagen, Germany) and QIAamp 96 DNA Buccal Swab BioRobot kit (Qiagen, Valencia, CA). For the South African samples, the *CYP3A4 *rs2740574 and *CYP3A5 *rs776746 polymorphisms were analyzed as previously described [27]. For the Senegalese samples, the *CYP3A4* rs2740574 and *CYP3A5* rs776746 were analyzed using the methods described previously [19]. The *SRD5A2* polymorphisms rs9282858 (A49T) and rs523349 (V89L) for both sets of samples were analyzed using the methods described by Zeigler-Johnson and colleagues [28].

### 2.4. Statistical Analysis

Analyses were undertaken to compare the association of prostate cancer, advanced tumor stage, and Gleason scores with the presence of androgen metabolism variants. Results were stratified by sample population. Descriptive analyses for discrete traits were carried out using contingency table methods and the Fisher's exact tests (FET) or chi-square statistics. Medians were used to summarize continuously distributed traits. Unconditional logistic regression was used to examine population differences in the odds of having advanced disease at diagnosis. Genotypes for each gene were analyzed in separate models using the homozygous wild type as the comparison group. All analyses were adjusted for age. Statistical heterogeneity among groups was tested using the Mantel-Haenszel test of independence. To minimize the effect of potential false-positive genetic associations due to the small sample sizes of the individual population groups, we combined the genotype data by country (combined South African versus Senegalese) and by non-Black versus Black (Mixed Ancestry/White versus South African Black/Senegalese) (due to the significant ancestral contributions of other populations, we categorized the Mixed Ancestry as non-Black). Previously, we corrected for population stratification in a South African Mixed Ancestry group and in a combined Mixed Ancestry/White group, by applying a genomic control inflation factor value, lambda (*λ*) [27, 29]. Given that *λ* may increase with sample size, in the present study we applied a more stringent correction for population stratification by using a calculated *λ*_1,000_ [30], an adjusted inflation factor assuming an equivalent study of 1,000 cases and 1,000 controls. Consequently, upon applying this correction for our association analyses, only observed *P* values of less than and equal to 0.0041 were considered statistically significant for the Mixed Ancestry and the combined Mixed Ancestry/White group; this *P* value was also applied when the Mixed Ancestry was combined with South African White and Black groups. A two-sided *P* value of 0.05 or less was considered statistically significant for the South African White, Black, Senegalese, and the combined South African Black/Senegalese group.

## 3. Results and Discussion

The median age at diagnosis of prostate cancer and the median age of the controls was significantly different among the groups (Table 1). Senegalese were the youngest for both controls (median age 50) and cases (median age 66). Median PSA level of Senegalese cases was higher than levels of South African Black, Mixed Ancestry, and White cases (57.5 ng/mL versus 47.8 ng/mL, 19.3 ng/mL, and 14.3 ng/mL) (Table 1). Previous studies also demonstrated that Senegalese men have higher PSA levels than African-American, Caucasian-American, and Asian-Indian men [13, 14], although the high PSA in Senegalese men does not appear to correlate with higher levels of aggressive disease. South African Black men showed the highest proportion of advanced disease (high-stage tumors T3/T4) (Table 1). Advanced disease could be linked to seeking potentially curative therapy at a later age when the disease may be at a much more advanced stage. However, Heyns and colleagues [12] and the present study showed the that South African Black men generally present with clinical features at a similar age to men from other groups. Therefore, these data might suggest that South African Black men are predisposed to develop more aggressive disease. Overall, metastasis was observed more often in Senegalese men than in South African men (16.3% versus 13.6% among Black South African, 10.7% Mixed Ancestry, and 3.0% White South African) (Table 1). A possible reason for this may be that the Senegalese men have a better follow-up schedule [13], whereas many South African men were lost to follow-up or the mean duration of follow-up was short, particularly for men with higher-stage and grade disease [12].

**Figure 1:**
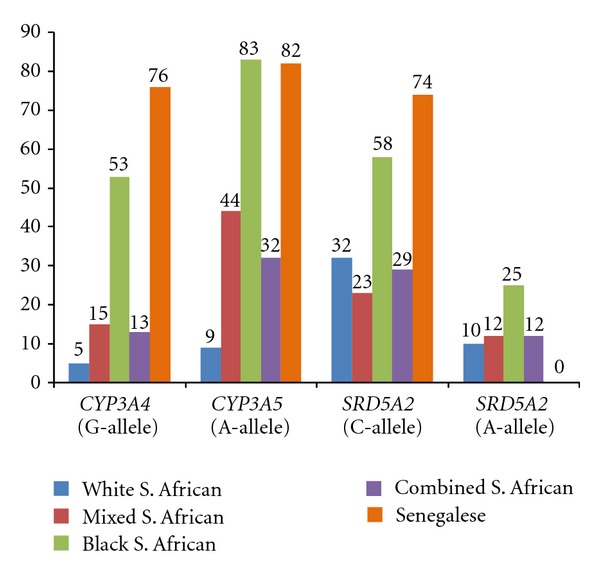
Minor allele frequencies among South African and Senegalese controls.

We noted significant genotype frequency differences between the South African ethnic groups, as well as between the South African and Senegalese populations (Table 2). There were significant differences in allele frequencies between the South African and Senegalese groups for *CYP3A4* and *SRD5A2* (Figure 1). Conversely, the frequencies for the *CYP3A5* rs776746 A-allele were not significantly different in South African Black and Senegalese men (Figure 1). These data might suggest that the diverse populations with African ancestry might share some common prostate cancer susceptibility alleles that may be different than the non-African populations. 

A positive association was observed between the *CYP3A4* rs2740574 AG and AG/GG genotypes and prostate cancer in South African White and Mixed Ancestry men and the *SRD5A2* rs9282858 AG and AG/AA genotypes in Mixed Ancestry men, whereas the *SRD5A2* rs523349 CC and CG/CC genotypes demonstrated an inverse association with the disease in Black South Africans (Table 3). When we combined the groups by country, only the *CYP3A4* rs2740574 association remained significant (Table 4). Combining the groups by non-Black versus Black, the association with the *CYP3A4* rs2740574 AG and AG/GG genotype was significant in the combined South African Mixed Ancestry/White group, and an inverse association was observed for the *SRD5A2* 523349 CG, CC and CG/CC genotypes in the combined South African Black/Senegalese group (Table 5). We consistently observed the associations with *CYP3A4* rs2740574 polymorphism, of which the variant G-allele has been shown to have no functional consequence [31]. Our data support numerous associations reported in other populations [17–19]. However, given that the polymorphism has no functional consequence, the *CYP3A4* rs2740574 associations reported in various populations are unlikely to be causal and might in fact reflect the influence of a nearby variant in linkage disequilibrium (LD). For *SRD5A2*, the variant A-allele of the rs9282858 polymorphism has been shown to cause the steroid 5-alpha reductase enzyme to have a higher Vmax compared to the wild-type enzyme [32]. Given that the *SDR5A2* rs9282858 association that we observed in Mixed Ancestry men did not remain significant when we combined different groups, we cannot exclude that this association could be spurious due to the small sample size of the individual groups or population stratification. Physiologically, the protective effects we observed for *SRD5A2* rs523349 in South African Black and the combined Black/Senegalese group are plausible because the variant C-allele, which causes a Valine to Leucine (V89L) amino acid substitution at codon 89, results in a reduced steroid 5-alpha reductase enzyme activity [33]. Given that the conversion of testosterone to highly active DHT may play an import role in prostate cancer development [34], it stands to reason that a significant reduction in steroid 5-alpha reductase activity may reduce the likelihood of developing prostate cancer.

Stratifying the genotype data according to pathological characteristics, an association was observed with the *CYP3A4* rs2740574 AG and AG/GG genotypes and an increased likelihood of developing high-stage and high-grade prostate cancer in Mixed Ancestry, the combined South African and the non-Black (Mixed Ancestry/White) group (data no shown). However, it remains unclear how a “nonfunctional” variant may alter tumor pathology. Additionally, Senegalese men were more than 0.16-fold (84%) less likely to develop high-stage prostate cancer if they had the *SRD5A2* rs523349 CG genotype (data not shown). These data might lend support for the use of steroid 5-alpha reductase inhibitors to limit progression to advanced disease. 

We observed a few possible limitations in our study. Our sample sizes for the individual groups were small (particularly in the Black South African group) and may thus have been underpowered to detect discrete genetic associations. However, we were sufficiently powered to observe statistical differences in the other groups, especially the mixed South African sample. We computed tests of heterogeneity to determine that there are indeed significant differences in the estimates that were obtained for the primary associations of interest. Specifically, there was significant heterogeneity among the estimates for *CYP3A4* rs2740574 (*P* < 0.001) and *SRD5A2 *rs9282858 (*P* = 0.021). Therefore, in those situations where we are unable to detect a significant association, we may still observe that populations differ from one another in how these genotypes are related to prostate cancer. There are other issues besides small sample size that may contribute to the heterogeneity in associations. Environmental/lifestyle differences among these populations may contribute to gene-environment effects causing differences in association. Linkage disequilibrium with other putative genes such as *CYP3A43* (not measured here, but on the same locus as *CYP3A4* and *CYP3A5*) may exist in some populations and not others [19]. In case-control association studies, unobserved population stratification also may act as a confounder, leading to an increased number of false-positive results. The South African Mixed Ancestry population exhibits significant population stratification [9]. We subjected our South African Mixed Ancestry group and the combined South African groups to stringent genomic control correction. This approach may have limitations and ideally, we should use ancestry informative markers (AIMs) to determine the level of substructure in the Mixed Ancestry group, then extrapolate these data to the combined groups. However, there are currently no available AIMs specific for the Mixed Ancestry population that we could use to directly measure the effect of substructure. For the other groups included in the study, we considered the admixture in the Senegalese population to be low [35], that, due to historical separation between South African Black and White population, admixture is also low, or at best, not to the extent seen in the Mixed Ancestry population.

## 4. Conclusions

We investigated the role of androgen metabolism gene polymorphisms in prostate cancer risk and tumor pathology in African men with diverse ancestries. We observed significant differences in allele frequencies between South African and Senegalese individuals. We also established that the allele frequencies were more similar between Senegalese and Black South African men. The magnitude and direction of the effects we observed for *CYP3A4* rs274057 were comparable to other studies. For *SRD5A2* rs523349, protective effects associated with tumor pathology were seen in Senegalese men, but not in South African men. Our results suggest that, variants previously associated with prostate cancer in other populations may also affect prostate cancer risk and tumor aggressiveness in African men. Given that ethnicity is considered a significant risk factor for prostate cancer, the comparative analyses in diverse African populations may help to elucidate the underlying genetic etiolgies of the disease. 

## Figures and Tables

**Table 1:** Description of clinical characteristics among South African and Senegalese prostate cancer cases and controls.

	South Africa						Senegal		
Variables of interest	White		Mixed		Black		Senegalese		*P*
	Controls (*n* = 134)	Cases (*n* = 120)	Controls (*n* = 167)	Cases (*n* = 207)	Controls (*n* = 20)	Cases (*n* = 25)	Controls (*n* = 300)	Cases (*n* = 86)	
^ a^Median age (years)	63	71	61	67	60	71	50	66	<0.001 (controls) 0.002 (cases)
^ b^Median PSA (ng/mL)	0.8	14.3	0.9	19.3	1.4	47.8	1.2	57.5	<0.001 (controls) <0.001 (cases)
^‡^Gleason ≥ 7		34/78 (43.6%)		62/130 (47.7%)		9/12 (75.0%)		34/76 (40.5)	0.146 (cases)
^‡^Stage 3/4		35/104 (33.7%)		85/203 (41.9%)		14/24 (58.3%)		35/78 (44.9%)	0.121 (cases)
^‡^Metastasis		3/100 (3.0%)		21/196 (10.7%)		3/22 (13.6%)		14/86 (16.3%)	0.003 (cases)

^a^For each of the respective population groups, *P* ≤ 0.05 when comparing the median age between cases versus controls.

^b^For each of the respective population groups, *P* ≤ 0.05 when comparing the median PSA between cases versus controls.

^‡^Gleason score, tumor stage and/or metastases information were missing from the medical records of some of the clinically confirmed prostate cancer cases. Therefore, the numbers and percentages shown are for the available information, and not that of the total number of cases included in the study for each population group.

**Table 2:** Frequency of genotypes among South African and Senegalese prostate cancer cases and controls.

	South Africa								Senegal	
Genotypes	White		Mixed		Black		Combined		Senegalese	
Genes	Controls (*n* = 134)	Cases (*n* = 120)	Controls (*n* = 167)	Cases (*n* = 207)	Controls (*n* = 20)	Cases (*n* = 25)	Controls (*n* = 321)	Cases (*n* = 352)	^‡^Controls (*n* = 300)	^‡^Cases (*n* = 86)
*CYP3A4* (rs2740574)										
AA	122 (91.0%)	84 (70.0%)	117 (70.1%)	82 (39.6%)	2 (10.0%)	9 (36.0%)	241 (75.1%)	175 (49.7%)	21 (7.5%)	6 (7.1%)
AG	12 (9.0%)	34 (28.3%)	49 (29.3%)	111 (53.6%)	15 (75.0%)	16 (64.0%)	76 (23.7%)	161 (45.7%)	91 (32.3%)	40 (47.6%)
GG	0 (0%)	2 (1.7%)	1 (0.6%)	14 (6.8%)	3 (15.0%)	0 (0%)	4 (1.2%)	16 (4.6%)	170 (60.3%)	38 (45.2%)

*CYP3A5* (rs776746)										
GG	113 (84.3%)	95 (79.2%)	58 (34.7%)	58 (28.0%)	1 (5.0%)	1 (4.0%)	172 (53.6%)	154 (43.8%)	14 (5.3%)	4 (5.1%)
AG	18 (13.4%)	22 (18.3%)	72 (43.1%)	106 (51.2%)	5 (25.0%)	5 (20.0%)	95 (29.6%)	133 (37.8%)	66 (25.1%)	20 (25.3%)
AA	3 (2.3%)	3 (2.5%)	37 (22.2%)	43 (20.8%)	14 (70.0%)	19 (76.0%)	54 (16.8%)	65 (18.4%)	183 (69.6%)	55 (69.6%)

*SRD5A2* V89L (rs523349)										
GG	56 (41.8%)	61 (50.8%)	96 (57.5%)	131 (63.3%)	5 (25.0%)	16 (64.0%)	157 (48.9%)	208 (59.1%)	26 (12.2%)	10 (14.1%)
CG	70 (52.2%)	51 (42.5%)	65 (38.9%)	71 (34.3%)	7 (35.0%)	7 (28.0%)	142 (44.2%)	129 (36.6%)	61 (28.5%)	10 (14.1%)
CC	8 (6.0%)	8 (6.7%)	6 (3.6%)	5 (2.4%)	8 (40.0%)	2 (8.0%)	22 (6.9%)	15 (4.3%)	127 (59.4%)	51 (71.8%)

*SRD5A2* A49T (rs9282858)										
GG	107 (79.8%)	90 (75.0%)	127 (76.0%)	120 (58.0%)	10 (50.0%)	17 (68.0%)	244 (76.0%)	227 (64.5%)	278 (100.0%)	75 (100.0%)
GA	27 (20.2%)	30 (25.0%)	40 (24.0%)	87 (42.0%)	10 (50.0%)	8 (32.0%)	77 (24.0%)	125 (35.5%)	0 (0%)	0 (0.0%)

*P* < 0.001, for each polymorphism when comparing across each of the respective population groups for cases or for controls.

^‡^Some participants had missing genotype data. The numbers and percentages shown are for the available information.

**Table 3:** South African and Senegalese case-control genotype associations with prostate cancer (adjusted for age).

Variables of interest	South Africa			Senegal
	White	Mixed	Black	Senegalese
Genotypes	OR (95% CI)	OR (95% CI)	OR (95% CI)	OR (95% CI)
*CYP3A4* (rs2740574)				
AA	1.00 (ref)	1.00 (ref)	1.00 (ref)	1.00 (ref)
AG	**3.32** (**1.52–7.24**)*	**3.38** (**2.13–5.35**)**	0.21 (0.03–1.32)	1.51 (0.52–4.38)
GG	—	4.57 (1.63–12.85)	—	0.94 (0.54–1.65)
AG/GG	**3.63** (**1.68–7.84**)*	**3.74 **(**2.37–5.89**)**	0.18 (0.03–1.16)	1.13 (0.40–3.22)

*CYP3A5* (rs776746)				
GG	1.00 (ref)	1.00 (ref)	1.00 (ref)	1.00 (ref)
AG	1.14 (0.55–2.37)	1.68 (1.02–2.76)	1.31 (0.36–29.51)	0.98 (0.18–5.39)
AA	1.14 (0.46–2.79)	1.21 (0.89–1.64)	1.65 (0.38–7.15)	0.99 (0.50–1.99)
AG/AA	1.17 (0.59–2.32)	1.61 (1.01–2.57)	2.21 (0.12–39.39)	0.98 (0.24–3.97)

*SRD5A2 *V89L (rs523349)				
GG	1.00 (ref)	1.00 (ref)	1.00 (ref)	1.00 (ref)
CG	0.71 (0.41–1.24)	0.74 (0.48–1.16)	0.26 (0.04–1.54)	0.36 (0.12–1.07)
CC	0.86 (0.49–1.50)	0.78 (0.41–1.48)	**0.17** (**0.04**–**0.66**)*	0.98 (0.60–1.59)
CG/CC	0.71 (0.41–1.22)	0.73 (0.47–1.13)	**0.14** (**0.03**–**0.66**)*	0.74 (0.30–1.86)

*SRD5A2* A49T (rs9282858)				
GG	1.00 (ref)	1.00 (ref)	1.00 (ref)	1.00 (ref)
AG	1.02 (0.54–1.95)	**2.31** (**1.45**–**3.68**)**	0.43 (0.11–1.69)	—
AA	—	—	—	—
AG/AA	1.02 (0.54–1.95)	**2.31** (**1.45**–**3.68**)**	0.43 (0.11–1.69)	—

—: too few/no genotypes to estimate ORs.

*P < 0.05.

**P < 0.0041.

**Table 4:** Combined South African and Senegalese case-control genotype associations with prostate cancer (adjusted for age).

Variables of interest	South Africa (Mixed, White and Black)	Senegal
Genotypes	OR (95% CI)	OR (95% CI)
*CYP3A4* (rs2740574)		
AA	1.00 (ref)	1.00 (ref)
AG	**3.04** (**2.13**–**4.35**)**	1.51 (0.52–4.38)
GG	3.11 (1.63–5.93)	0.94 (0.54–1.65)
AG/GG	**3.27** (**2.30**–**4.65**)**	1.13 (0.40–3.22)

*CYP3A5* (rs776746)		
GG	1.00 (ref)	1.00 (ref)
AG	1.74 (1.21–2.51)	0.97 (0.18–5.39)
AA	1.33 (1.05–1.67)	0.99 (0.50–1.99)
AG/AA	1.74 (1.25–2.41)	0.98 (0.24–3.97)

*SRD5A2* V89L (rs523349)		
GG	1.00 (ref)	1.00 (ref)
CG	0.66 (0.47–0.92)	0.36 (0.12–1.07)
CC	0.66 (0.45–0.95)	0.98 (0.60–1.59)
CG/CC	0.62 (0.45–0.86)	0.74 (0.30–1.86)

*SRD5A2* A49T (rs9282858)		
GG	1.00 (ref)	1.00 (ref)
AG	1.70 (1.19–2.43)	—
AA	—	—
AG/AA	1.70 (1.19–2.43)	—

—: too few/no genotypes to estimate ORs.

**P < 0.0041.

**Table 5:** Case-control genotype associations with prostate cancer by non-Black and Black men (adjusted for age).

Variables of interest	Non–Black (South African Mixed and White)	Black (South African Black and Senegalese)
Genotypes	OR (95% CI)	OR (95% CI)
*CYP3A4* (rs2740574)		
AA	1.00 (ref)	1.00 (ref)
AG	**3.58** (**2.44**–**5.24**)**	0.80 (0.35–1.82)
GG	5.16 (1.85–14.39)	0.65 (0.41–1.02)
AG/GG	**3.95** (**2.72**–**5.75**)**	0.59 (0.26–1.31)

*CYP3A5* (rs776746)		
GG	1.00 (ref)	1.00 (ref)
AG	1.78 (1.23–2.58)	1.06 (0.24–4.63)
AA	1.30 (1.00–1.69)	1.08 (0.57–2.02)
AG/AA	1.75 (1.25–2.45)	1.13 (0.32–3.98)

*SRD5A2* V89L (rs523349)		
GG	1.00 (ref)	1.00 (ref)
CG	0.68 (0.48–0.96)	**0.26** (**0.11**–**0.61**)*
CC	0.79 (0.52–1.20)	**0.67** (**0.46**–**0.97**)*
CG/CC	0.67 (0.48–0.94)	**0.38** (**0.19**–**0.77**)*

*SRD5A2* A49T (rs9282858)		
GG	1.00 (ref)	1.00 (ref)
AG	1.88 (1.30–2.73)	1.40 (0.48–4.12)
AA	—	—
AG/AA	1.88 (1.30–2.73)	1.40 (0.48–4.12)

—: too few/no genotypes to estimate ORs.

*P < 0.05.

**P < 0.0041.
